# The association between attention deficit hyperactivity disorder and pregnancy, delivery and neonatal outcomes—an evaluation of a population database

**DOI:** 10.1186/s12884-024-06561-5

**Published:** 2024-05-15

**Authors:** Uri Amikam, Ahmad Badeghiesh, Haitham Baghlaf, Richard Brown, Michael H. Dahan

**Affiliations:** 1 Department of Obstetrics and Gynecology, McGill University, 845 Rue Sherbrooke, O, Montréal, QC 3HA 0G4 Canada; 2 Faculty of Medicine, Tel Aviv University, Tel Aviv, Israel; 3 Department of Obstetrics and Gynecology, King Abdulaziz University, Rabigh Branch, Rabigh, Saudi Arabia; 4 Department of Obstetrics and Gynecology, University of Tabuk, Tabuk, Saudi Arabia

**Keywords:** Attention deficit hyperactivity disorder, Neuropsychiatric disorder, Maternal morbidity, Hypertensive disorders of pregnancy, Perinatal outcomes, Cesarean delivery, Maternal infection, Small-for-gestational-age

## Abstract

**Background:**

Attention deficit hyperactivity disorder (ADHD) is one of the more common neuropsychiatric disorders in women of reproductive age. Our objective was to compare perinatal outcomes between women with an ADHD diagnosis and those without.

**Methods:**

A retrospective population-based cohort study utilizing the Healthcare Cost and Utilization Project, Nationwide Inpatient Sample (HCUP-NIS) United States database. The study included all women who either delivered or experienced maternal death from 2004 to 2014. Perinatal outcomes were compared between women with an ICD-9 diagnosis of ADHD and those without.

**Results:**

Overall, 9,096,788 women met the inclusion criteria. Amongst them, 10,031 women had a diagnosis of ADHD. Women with ADHD, compared to those without, were more likely to be younger than 25 years of age; white; to smoke tobacco during pregnancy; to use illicit drugs; and to suffer from chronic hypertension, thyroid disorders, and obesity (*p* < 0.001 for all). Women in the ADHD group, compared to those without, had a higher rate of hypertensive disorders of pregnancy (HDP) (aOR 1.36, 95% CI 1.28–1.45, *p* < 0.001), cesarean delivery (aOR 1.19, 95% CI 1.13–1.25, *p* < 0.001), chorioamnionitis (aOR 1.34, 95% CI 1.17–1.52, *p* < 0.001), and maternal infection (aOR 1.33, 95% CI 1.19–1.5, *p* < 0.001). Regarding neonatal outcomes, patients with ADHD, compared to those without, had a higher rate of small-for-gestational-age neonate (SGA) (aOR 1.3, 95% CI 1.17–1.43, *p* < 0.001), and congenital anomalies (aOR 2.77, 95% CI 2.36–3.26, *p* < 0.001).

**Conclusion:**

Women with a diagnosis of ADHD had a higher incidence of a myriad of maternal and neonatal complications, including cesarean delivery, HDP, and SGA neonates.

**Supplementary Information:**

The online version contains supplementary material available at 10.1186/s12884-024-06561-5.

## Background

Attention deficit hyperactivity disorder (ADHD) is one of the most common neuropsychiatric disorders in childhood and adolescence [1]. Estimates suggest around 5% of children globally are affected with persistence as a chronic condition into adulthood in half of these, representing about 2,6% of adults [1–5]. Over recent decades there has been a significant increase in the prevalence of children diagnosed with ADHD, and this increase has been observed in both genders [6]. The diagnosis of ADHD is based upon the Psychiatric Association’s Diagnostic and Statistical Manual of Mental Disorders, Fifth edition (DSM-5) [7] and is based on the presence of pervasive, developmentally excessive, and impairing levels of impulsivity, overactivity, and inattention. There are mainly three subtypes of ADHD, including primarily inattentive, primarily hyperactive-impulsive, or combined. The term attention deficit disorder (ADD) was first introduced in the third edition of the DSM (DSM-III), and two subtypes of ADD were identified (ADD with hyperactivity and without). Later on, a revised version of DSM (DSM-III-R), and the term ADHD was first introduced with the elimination of ADD [8]. Although ADD was removed from the DSM diagnosis, it is still used in practice interchangeably with ADHD and has a specific ICD-9 code.

ADHD in adulthood has several associations, including serving as a risk factor for a diverse array of mental health issues, encompassing defiant, disruptive, and antisocial behaviors, emotional challenges, self-harm, and substance misuse [1]. It is also associated with broader negative outcomes, such as educational underachievement, difficulties in employment and relationships, and involvement in criminal activities [1]. It can be speculated that all these varied impairments could potentially result in adverse effects on pregnancy.

To date, data on perinatal outcomes in women with an ADHD diagnosis is relatively limited, and previous studies had several limitations, including missing important characteristics such as body mass index (BMI), illicit drug use, and socioeconomic status [4, 9] and delivery outcomes such as mode of delivery and birthweight categorization [4].

Given the paucity of data in the medical literature, our objective was to assess maternal and neonatal outcomes in women with ADHD using a comprehensive contemporary nationwide database.

## Methods

This study was a retrospective population-based cohort study, utilizing data from the Healthcare Cost and Utilization Project Nationwide Inpatient Sample (HCUP-NIS). HCUP-NIS, the largest inpatient sample database in the USA, comprises hospital inpatient stays submitted by facilities across nearly the entire country. The data is representative of 20% of admissions to US hospitals across 48 states and the District of Columbia, sourced from approximately 1000 hospitals. The cohort consisted of pregnant women delivering between 2004–2014, and it was confined to admissions resulting in delivery or maternal death, ensuring individuals were included only once per pregnancy in the assessment.

Women diagnosed with ADD or ADHD constituted the study group, while women without these diagnoses constituted the control group. We combined these two diagnoses given their interchangeable use in the literature.

Patients’ ADD/ADHD status was categorized based on an International Classification of Disease, Ninth Revision, Clinical Modification (ICD-9-CM) diagnosis of ADHD and ADD, which included the codes 314.00 and 314.01, respectively.

The collected data encompassed demographic and obstetric parameters and details on the labor process, as well as short-term maternal and neonatal outcomes up to the point of discharge. Demographic parameters included maternal age, race, income quartiles, and the type of insurance. Medicare is federal health insurance for people 65 years of age or older, and some people under 65 with certain disabilities or conditions; whilst Medicaid is a joint federal and state program that helps cover medical costs for some people with limited income and resources. Labor and delivery parameters included hypertensive disorders of pregnancy (HDP) (which included any of the following: gestational hypertension, eclampsia, and preeclampsia); gestational diabetes mellitus (GDM); placenta previa; preterm delivery (PTD) (< 37 weeks); preterm premature rupture of membranes; cesarean delivery (CD); wound complications; postpartum hemorrhage (PPH); maternal infection; venous thromboembolism; deep vein thrombosis; pulmonary embolism; and disseminated intravascular coagulation. Neonatal outcomes investigated included: congenital anomalies; intra-uterine fetal death (IUFD); and small-for-gestational-age (SGA) neonates.

### Statistical analysis

An initial analysis was conducted to determine the prevalence of women with an ADHD diagnosis over the entire study duration. Following that, chi-square tests were employed to compare the baseline characteristics between women with an ADHD diagnosis and those without. Logistic regression analyses were subsequently conducted to evaluate effects of an ADHD diagnosis on maternal and neonatal outcomes. The adjusted regression models controlled for potential confounding effects, including maternal demographics, pre-existing clinical characteristics, and concurrently occurring conditions that had achieved significance (*p* < 0.05) on the chi-squared tests in the initial analysis (Table 1). Additionally, the Bonferroni correction was used to control for the increased risk of Type I errors given the number of hypotheses tested in our study. All analyses were performed using SPSS 25.0 (IBM Corporation, Chicago, USA).

**Table 1:** Maternal characteristics

Characteristics	Attention deficit disorder *N* = 10,031	No Attention deficit disorder *N* = 9,086,757	*P*-value
Age (years)			** < 0.001**
< 25	5,827 (58.1%)	3,450,028 (38%)	
25–34	3,357 (33.5%)	4,296,546 (47.3%)	
≥ 35	847 (8.4%)	1,340,173 (14.7%)	
Race			** < 0.001**
White	7,508 (74.8%)	4,475,151 (49.2%)	
Black	1,398 (13.9%)	1,647,986 (18.1%)	
Hispanic	733 (7.3%)	2,028,595 (22.3%)	
Asian and Pacific	105 (1%)	441,710 (4.9%)	
Native American	56 (0.6%)	64,092 (0.7%)	
Other	198 (2%)	365,462 (4%)	
Income quartiles			** < 0.001**
Less than 39,000	2,457 (24.5%)	2,215,683 (24.4%)	
$39,000–47,999	3,135 (31.3%)	3,076,995 (33.9%)	
$48,000–62,999	2,614 (26.1%)	2,413,522 (26.6%)	
$63,000 or more	1,825 (18.2%)	1,1380,505 (15.2%)	
Plan type			** < 0.001**
Medicare	348 (3.5%)	56,255 (0.6%)	
Medicaid	5,213 (52%)	3,877,563 (42.7%)	
Private including HMO	3,961 (39.5%)	4,603,012 (50.7%)	
Self-pay	157 (1.6%)	288,279 (3.2%)	
No charge	< 11	17,058 (0.2%)	
Other	348 (3.5%)	244,590 (2.7%)	
Obesity (BMI ≥ 30 kg/m^2^)	891 (8.9%)	323,285 (3.6%)	** < 0.001**
Previous CD	1281 (12.8%)	1,451,209 (16%)	** < 0.001**
Tobacco Smoking during pregnancy	2,402 (23.9%)	441,188 (4.9%)	** < 0.001**
Chronic hypertension	257 (2.6%)	164,973 (1.8%)	** < 0.001**
Pregestational DM	156 (1.6%)	86,459 (1%)	** < 0.001**
Illicit drug use	954 (9.5%)	124,665 (1.4%)	** < 0.001**
Multiple gestation	155 (1.5%)	137,148 (1.5%)	0.768
Thyroid disorders	456 (4.5%)	222,822 (2.5%)	** < 0.001**
HIV	< 11	2075 (0%)	0.259
IVF	< 11	10,527 (0.1%)	0.151

Per convention of the HCUP database, when *N* < 11, absolute cell number of subjects was not provided to protect patient anonymity

*Abbreviations and definitions*: *HMO* Health Maintenance Organization, *BMI* Body Mass Index, *CD* cesarean delivery, *DM* diabetes mellitus, *HIV* human immunodeficiency virus, *IVF* in-vitro fertilization

Informed consent was waived due to the retrospective design of the study.

This study exclusively utilized publicly accessible, anonymized data, and as per articles 2.2 and 2.4 of the Tri-Council Policy Statement (2010) [10], institutional review board approval was deemed unnecessary.

## Results

**Fig. 1:**
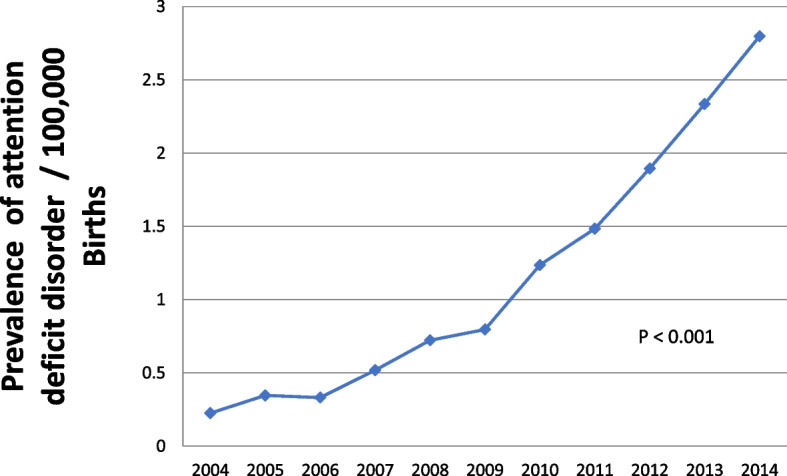
Prevalence of attention deficit hyperactivity disorder in pregnant women during the study period

A total of 9,096,788 women fulfilled the criteria for inclusion. Of them, 10,031 patients had a diagnosis of ADHD. Notably, the prevalence of ADHD diagnosis in the cohort increased significantly during the study period (*p* < 0.001), as depicted in Fig. 1.

The demographic and baseline characteristics of women with and without an ADHD diagnosis are listed in Table 1. Women with ADHD, compared to those without, were younger; more likely to be white; were wealthier; more likely to have Medicare or Medicaid health insurance; more likely to be obese (BMI ≥ 30 kg/m^2^); more likely to have previous CD; more inclined to engage in illicit drug use or tobacco smoking during pregnancy; and were more likely to have pre-existing chronic hypertension, thyroid disorders and pregestational diabetes mellitus (DM) (*p* < 0.001 for all). Other maternal characteristics, such as the rate of in-vitro fertilization conceptions and multiple gestations, were similar between the two groups.

Table 2 presents the association between ADHD and pregnancy and delivery outcomes, both before and after adjusting for potential confounders, including maternal age, race, insurance plan type, income quartiles, illicit drug use during pregnancy, tobacco smoking, obesity, previous CD, chronic hypertension, thyroid disorders and pregestational DM, with the addition of pregnancy-induced hypertension, gestational hypertension, preeclampsia, preeclampsia and eclampsia superimposed hypertension, and placenta previa for the analysis of delivery and neonatal outcomes. Women with an ADHD diagnosis, compared to those without, had a higher rate of HDP (adjusted OR (aOR) 1.24, 95%CI 1.13–1.36, *p* < 0.001); placenta previa (aOR 1.32, 95%CI 1.02–1.7, *p* = 0.032); chorioamnionitis (aOR 1.34, 95%CI 1.17–1.52, *p* < 0.001); CD (aOR 1.19, 95%CI 1.13–1.25,* p* < 0.001); PPH (aOR 1.23, 95%CI 1.11–1.37, *p* < 0.001); wound complications (aOR 1.51, 95%CI 1.17–1.96, *p* = 0.002); and maternal infection (aOR 1.33, 95%CI 1.19–1.5, *p* < 0.001). The details on the different components of HDP are presented in Supplementary Table 1. Notably, after employing the Bonferroni correction, placenta previa did not reach statistical significance (data not shown in the tables).

**Table 2:** Pregnancy and delivery outcomes

Outcomes	Attention deficit disorder (%)	No Attention deficit disorder (%)	Crude OR (95% CI)	Adjusted OR (95% CI)	Adjusted *p*-value
**Pregnancy Outcomes**^a^					
HDP	1,092 (10.9%)	672,657 (7.4%)	1.53 (1.44–1.63)	1.36 (1.28–1.45)	< 0.001
GDM	497 (5%)	522,695 (5.8%)	0.85 (0.78–0.94)	1.04 (0.95–1.14)	0.36
Placenta previa	61 (0.6%)	49,921 (0.5%)	1.1 (0.86–1.43)	1.32 (1.02–1.7)	0.032
**Delivery Outcomes**^b^					
PPROM	150 (1.5%)	103,468 (1.1%)	1.32 (1.12–1.55)	1.15 (0.98–1.35)	0.094
Preterm delivery	902 (9%)	652,993 (7.2%)	1.28 (1.19–1.37)	1.05 (0.98–1.12)	0.205
Abruptio placenta	142 (1.4%)	97,337 (1.1%)	1.33 (1.12–1.57)	0.99 (0.84–1.17)	0.92
Chorioamnionitis	236 (2.4%)	165,094 (1.8%)	1.3 (1.14–1.48)	1.34 (1.17–1.52)	< 0.001
Operative vaginal delivery	516 (5.1%)	488,885 (5.4%)	0.95 (0.87–1.04)	0.96 (0.86–1.04)	0.265
CD	3,411 (34%)	2,936,507 (32.3%)	1.08 (1.04–1.13)	1.19 (1.13–1.25)	< 0.001
SVD	6,104 (60.9%)	5,661,365 (62.3%)	0.94 (0.9–0.98)	0.87 (0.84–0.91)	< 0.001
Hysterectomy	< 11	7,092 (0.1%)	0.9 (0.43–1.88)	0.95 (0.44–2.02)	0.883
PPH	365 (3.6%)	263,600 (2.9%)	1.26 (1.14–1.4)	1.23 (1.11–1.37)	< 0.001
Wound complications	59 (0.6%)	32,674 (0.4%)	1.64 (1.27–2.12)	1.51 (1.17–1.96)	0.002
Maternal Death	< 11	636 (0%)	2.85 (0.71–11.42)	2.69 (0.67–10.83)	0.165
Transfusion	118 (1.2%)	90,249 (1%)	1.19 (0.99–1.43)	1.06 (0.88–1.28)	0.524
**Others**					
Maternal infection	286 (2.9%)	198,982 (2.2%)	1.31 (1.17–1.48)	1.33 (1.19–1.5)	< 0.001
DVT	< 11	3,826 (0%)	1.42 (0.64–3.17)	1.24 (0.55–2.76)	0.604
PE	< 11	1,656 (0%)	1.64 (0.53–5.01)	1.31 (0.42–4.09)	0.637
VTE	< 11	5,301 (0.1%)	1.54 (0.8–2.96)	1.3 (0.68–2.51)	0.429
DIC	22 (0.2%)	18,222 (0.2%)	1.09 (0.72–1.66)	1.06 (0.7–1.62)	0.771

Per convention of the HCUP database when N < 11, absolute cell number of subjects was not provided to protect patient anonymity

*Abbreviations and definitions*: *HDP* hypertensive disorders of pregnancy, *GDM* gestational diabetes mellitus, *PPROM* preterm premature rupture of membranes, *CD* cesarean delivery, *SVD* spontaneous vaginal delivery, *PPH* post-partum hemorrhage, *DVT* deep vein thrombosis, *PE* pulmonary embolism, *VTE* venous thromboembolism, *DIC* disseminated intravascular coagulation

^a^Pregnancy Outcomes: Adjusted for age, race, plan type, income quartiles, illicit drug use, chronic hypertension, tobacco smoking during pregnancy, obesity, previous CD, thyroid disorders, and pregestational-DM

^b^Delivery Outcomes: Adjusted for age, race, plan type, income quartiles, illicit drug use, chronic hypertension, tobacco smoking during pregnancy, obesity, previous CD, thyroid disorders, pregestational DM, placenta previa, and HDP

Notably, although PTD rates were increased in women with ADHD in the non-controlled comparison, this lost statistical significance when controlling for confounders. Other pregnancy and delivery outcomes examined, such as GDM, placental abruption, and maternal death, were similar between the groups.

Neonatal outcomes are presented in Table 3. Women with a diagnosis of ADHD, compared to those without, had a higher rate of SGA neonates (aOR 1.3, 95%CI 1.17–1.43, *p* < 0.001); and congenital anomalies (aOR 2.77, 95%CI 2.36–3.26, *p* < 0.001). These findings remained statistically significant after performing the Bonferroni correction. There was no difference in the rate of IUFD between the two groups.

**Table 3:** Neonatal outcomes^a^

Outcomes	Attention deficit disorder (%)	No Attention deficit disorder (%)	Crude OR (95% CI)	Adjusted OR (95% CI)	Adjusted *p*-value
SGA	410 (4.1%)	197,660 (2.2%)	1.92 (1.74–2.12)	1.3 (1.17–1.43)	< 0.001
IUFD	41 (0.4%)	38,218 (0.4%)	0.97 (0.72–1.32)	0.85 (0.63–1.16)	0.307
Congenital Anomalies	149 (1.5%)	38,095 (0.4%)	3.58 (3.05–4.21)	2.77 (2.36–3.26)	< 0.001

*Abbreviations and definitions*: *SGA* small for gestational age, *IUFD* intrauterine fetal death

^a^Adjusted for age, race, plan type, income quartiles, illicit drug use, chronic hypertension, tobacco smoking during pregnancy, obesity, previous CD, thyroid disorders, pregestational DM, HDP, and placenta previa

## Discussion

We compared perinatal outcomes between women with an ADHD diagnosis and those without. We identified an increasing prevalence in ADHD diagnoses among pregnant women across the study period. Women with ADHD were more likely to be younger, of white race, in the higher income quartiles, to have Medicare or Medicaid insurance type, and had higher rates of obesity, previous CD, chronic hypertension, thyroid disorders, pregestational DM, tobacco smoking, and illicit drug use during pregnancy. Women with ADHD had an increased risk for CD, hypertensive disorders of pregnancy (HDP), chorioamnionitis, maternal infection, wound complications and PPH, and in additionally their infants had increased rates of being SGA and of having congenital anomalies.

Across the 10-year study period, there was a significant increase in the prevalence of an ADHD diagnosis amongst pregnant women (*p* < 0.001). This corresponds to the rising incidence of ADHD diagnosis amongst children and adolescents in the US reported between 1997–2016 [6]. These increases could represent either a true rise in the actual prevalence of ADHD over time or increased rates of diagnosis perhaps due to improved recognition of underlying ADHD. Regardless of the reason for the increase, this finding underscores the need for robust evidence regarding the interactions between ADHD and pregnancy.

Women in the ADHD group were more likely to be younger and of white race which aligns with previous data that demonstrated that childhood conduct and hyperactive disorders were independently and significantly associated with becoming a teenage mother [11] and that the incidence in children is higher in those who are white compared to other races [12]. The higher incidence of ADHD diagnosis amongst white women could reflect racial and ethnic disparities and accessibility to mental health care rather than true racial differences. Previous studies have shown that racial minorities have been diagnosed with ADHD at lower rates than white individuals [13–15], stressing the importance of considering cultural influences on healthcare seeking and delivery, along with an increased understanding of the various social, psychological, and biological factors among different racial and ethnic groups [15].

We found higher rates of obesity, chronic hypertension, and pregestational DM in the ADHD group. The association between obesity and ADHD is well-documented, with previous studies suggesting genetic and environmental factors underlying the association [16, 17]. Similarly, the association between hypertension and ADHD is well-established [18, 19], with one study demonstrating a higher incidence of chronic hypertension and pregestational DM among adult women with ADHD [19].

Jones et al. examined ADHD symptoms in pregnant women, and how these symptoms may influence health behaviors [20], identifying that hyperactivity was significantly linked with smoking, caffeine, decreased prenatal vitamin use, and physical strain. Similarly, our cohort of ADHD mothers demonstrated higher rates of tobacco smoking and illicit drug use. Additionally, ADHD is a significant risk factor for the later development of substance-use disorders and cigarette smoking in both sexes [21]. These findings highlight the significance of screening for harmful prenatal habits in these patients and addressing them as indicated during the prenatal period. Perhaps in part associated with such behaviors, women with ADHD were found to have more unplanned pregnancies compared to controls [22], which this also being an independent risk factor for adverse pregnancy outcomes [23], including preeclampsia [24].

Women with ADHD had increased rates of HDP (excluding eclampsia), even after adjusting for potential confounders such as obesity, chronic hypertension, and pregestational DM. Poulton et al. found that women who received treatment for ADHD at least 1 year before, but not during, pregnancy had a higher risk of preeclampsia compared to controls, with an OR of 1.2 [9]. However, exposure during pregnancy was uncertain as this group consisted of women receiving the drug within a year of the pregnancy and did not confirm treatment throughout the pregnancy, furthermore, this latter group was small impacting the significance of these findings. Walsh et al. also found a higher risk of developing gestational hypertension in women with ADHD (OR of 1.3) [4]. Possible explanations for this association include pharmacotherapy and unplanned pregnancies. Psychostimulants, including methylphenidate or amphetamine derivatives, used in the treatment of ADHD have been found to increase the risk for preeclampsia [25]. Unfortunately, previous studies have limited data relating to the timing of drug exposures and the HCUP database does not link medication use with the subjects.

There was a significantly higher rate of CD in the ADHD group, as previously demonstrated [9]. There may be various etiologies behind this and at least perhaps in part, this is also linked with the higher rates of PPH that persist even after controlling for potential confounders, including placenta previa and HDP. The increased incidence of chorioamnionitis seen in these patients may also be contributory given the association of chorioamnionitis and dysfunctional uterine muscle contraction subsequent to inflammation [26]. The risk for PPH in women with ADHD was not examined in previous studies [4, 9, 22].

Infectious complications overall were more prevalent in the ADHD group, including chorioamnionitis, maternal infection, and wound complications. While perinatal infection was not assessed in the previous studies, there is evidence that women with ADHD are at higher risk of acquiring sexually transmitted infections (STIs) [27], which may lead to chorioamnionitis. Whether an ADHD diagnosis justifies increased surveillance and screening for STIs before and during pregnancy remains to be determined.

Amongst the neonates, we found higher rates of SGA and congenital anomalies in the ADHD group. Although not observed in a prior study [9], there are several plausible explanations for our finding. Unintended pregnancy, as was more frequently observed in the ADHD cohort is associated with low birthweight [23], whilst in addition related factors such as increased rates of alcohol consumption [22], also affect neonatal birthweight and increase the risk for congenital anomalies. Lastly, some of the stimulants, such as methylphenidate and amphetamine, have been found to be associated with higher risks for congenital anomalies [28]. Again, information on alcohol use in pregnancy and medication use is unavailable in the HCUP database.

There are several limitations to our study. Firstly, the database used does not contain information on medical treatments received by patients during pregnancy, which could independently affect pregnancy outcomes as noted above. The missing data on ADHD pharmacotherapy during the pregnancy limits ascertaining if the association we found between ADHD and adverse pregnancy outcomes is related to the pathophysiology of ADHD itself or to the medication used to treat this condition. It is worth mentioning a previous study from Australia [9] that showed that women with ADHD had adverse pregnancy outcomes even if they did not receive any treatment for ADHD during pregnancy. Another limitation of our study is that data on certain maternal characteristics were unavailable, including alcohol use during pregnancy and the presence of coexisting conditions such as depression, known to be associated with an ADHD diagnosis [22]. Additionally, due to the anonymized nature of the study, we couldn't ascertain whether a patient had more than one delivery during the study period. Finally, our cohort was restricted to the period before 2015 because later data had differential coding within HCUP, using ICD-10 codes which are incompatible with ICD-9 codes.

Nevertheless, our study possesses several strengths. Firstly, with our large cohort across a wide study period of 11 years, our study was sufficiently powered to detect differences between groups. Secondly, since our data are derived from a population-based cohort, the findings hold general applicability to the US population and can also offer insights for other societies. Lastly, we were able to examine a broad range of pregnancy and delivery complications, offering detailed insights that can empower physicians to provide more precise counseling to pregnant women diagnosed with ADHD.

We suggest that future studies should focus on whether closer follow-up, including cervical length monitoring and screening for STIs, can reduce the elevated incidence of PTDs observed in the ADHD group. Additionally, research should explore if other potential risk factors, such as alcohol consumption, which may be more prevalent in the ADHD group, could influence the findings of our study. Furthermore, it is worth investigating whether the higher incidence of SGA in the ADHD group was due to fetal growth restriction in these patients. Lastly, it would be valuable to examine the impact of commonly used medications for ADHD, such as methylphenidate, on the adverse outcomes observed among pregnant women with ADHD. In conclusion, women diagnosed with ADHD are at increased risk of obstetrical complications, such as HDP, CD and PPH, along with an elevated risk of delivering SGA neonates, and a higher risk for congenital anomalies. These results highlight the importance of comprehensive patient counseling, screening for hazardous behaviors, and multidisciplinary care provided by both obstetricians and psychiatrists, along with vigilant obstetric monitoring for individuals with ADHD throughout their pregnancies.

### Supplementary Information

Supplementary material 1.

## Data Availability

The study used the Healthcare Cost and Utilization Project (HCUP) database (https://hcup-us.ahrq.gov/db/nation/nis/nisdbdocumentation.jsp). The analyses used during the current study are available from the corresponding author upon reasonable request.

## Abbreviations

ADHD: Attention deficit hyperactivity disorder
HCUP-NIS: Healthcare Cost and Utilization Project, Nationwide Inpatient Sample
HDP: Hypertension disorders of pregnancy
SGA: Small-for-gestational-age
DSM: Diagnostic and Statistical Manual of Mental Disorders
ADD: Attention deficit disorder
BMI: Body mass index
ICD-9: International Classification of Diseases 9th Revision
GDM: Gestational diabetes mellitus
PTD: Preterm delivery
CD: Cesarean delivery
PPH: Postpartum hemorrhage
IUFD: Intra-uterine fetal death
OR: Odds ratio
CI: Confidence intervals
DM: Diabetes mellitus
STI: Sexually transmitted infection
