# Supplementary material for: The association between attention deficit hyperactivity disorder and pregnancy, delivery and neonatal outcomes—an evaluation of a population database
Source: BMC Pregnancy Childbirth. 2024 May 15;24:364. doi: 10.1186/s12884-024-06561-5 (PMC11095018; doi:10.1186/s12884-024-06561-5)
Supplement: Supplementary file 1 — Supplementary material 1. [file 12884_2024_6561_MOESM1_ESM.docx]

| Outcomes | Attention deficit disorder  (%) | No Attention deficit disorder  (%) | Adjusted  p-value |
| --- | --- | --- | --- |
| HDP | 1,092 (10.9%)^b^ | 672,657 (7.4%)^c^ | <0.001 |
| Gestational hypertension | 464 (4.6%) | 301,143 (3.3%) | <0.001 |
| Preeclampsia | 553 (5.5%) | 326,837 (3.6%) | <0.001 |
| Eclampsia | 12 (0.1%) | 6932 (0.1%) | 0.116 |
| Superimposed preeclampsia and eclampsia | 85 (0.8%) | 47,280 (0.5%) | <0.001 |

**Supplementary Table 1** Hypertensive Disorders of Pregnancy

1. 22 women had more than one diagnosis.
2. 9,535 women had more than one diagnosis.

Abbreviations and definitions: HDP – hypertensive disorders of pregnancy.
